# Efficient Reconstruction of Heterogeneous Networks from Time Series via Compressed Sensing

**DOI:** 10.1371/journal.pone.0142837

**Published:** 2015-11-20

**Authors:** Long Ma, Xiao Han, Zhesi Shen, Wen-Xu Wang, Zengru Di

**Affiliations:** School of Systems Science, Beijing Normal University, Beijing, 100875, P.R. China; Tianjin University, CHINA

## Abstract

Recent years have witnessed a rapid development of network reconstruction approaches, especially for a series of methods based on compressed sensing. Although compressed-sensing based methods require much less data than conventional approaches, the compressed sensing for reconstructing heterogeneous networks has not been fully exploited because of hubs. Hub neighbors require much more data to be inferred than small-degree nodes, inducing a cask effect for the reconstruction of heterogeneous networks. Here, a conflict-based method is proposed to overcome the cast effect to considerably reduce data amounts for achieving accurate reconstruction. Moreover, an element elimination method is presented to use the partially available structural information to reduce data requirements. The integration of both methods can further improve the reconstruction performance than separately using each technique. These methods are validated by exploring two evolutionary games taking place in scale-free networks, where individual information is accessible and an attempt to decode the network structure from measurable data is made. The results demonstrate that for all of the cases, much data are saved compared to that in the absence of these two methods. Due to the prevalence of heterogeneous networks in nature and society and the high cost of data acquisition in large-scale networks, these approaches have wide applications in many fields and are valuable for understanding and controlling the collective dynamics of a variety of heterogeneous networked systems.

## Introduction

Complex networks are the backbone of many complex systems and play a significant role in a variety of collective dynamics of complex systems [1–6]. However, a great challenge arises when many complex networks are directly measured because of limited technology. Thus, the need arises for addressing the inverse problem of complex networks, i.e., indirectly reconstructing complex networks from measurable data. This has been a fundamental problem of exploring complex networked systems, and the significance of the inverse problem has been increasingly recognized. Many approaches have been developed to reconstruct complex networks [7–10]. In particular, a series of network reconstruction methods based on compressed sensing theory [11–16] have recently been proposed [17–20]. The approaches exploit the natural sparsity of real complex networks and convert the network reconstruction problem into a sparse signal reconstruction problem that can be solved by compressed sensing algorithm from a small amount of data. The approaches have been applied to reconstructing epidemic spreading networks [19], coupled oscillator networks [10, 17], social interaction networks [18, 20], as well as communication and transportation networks [20]. Specifically, to implement the compressed sensing based method, one needs to decompose the task of reconstructing the entire network into inferring local structures centered at each node. The amount of data required for reconstructing a local structure of a node is determined by the number of node links. In general, more data are required to reconstruct a node with more links. Note that data can be shared by all nodes for inferring their local structures, indicating that the data for accurate reconstruction is determined by the nodes with maximum degrees. Thus, the maximum-degree node becomes the “cask short board” of reconstruction. The cask principle is not serious for a network with homogeneous degree distribution because all nodes with homogeneous degrees require similar data. By contrast, a serious problem arises for heterogeneous networks, especially for networks with a small fraction of hubs [21–25], such as scale-free networks [26, 27]. The amount of data for reconstructing the local structure of a hub will be much larger than the other nodes. Many data measurements are merely used for a few nodes, accounting for the inefficiency of implementing this method. Thus, a practically important problem is raised: is it possible to reduce the data amount for reconstructing heterogeneous networks? Another situation that if often encountered and likely reduces the required data is the presence of a fraction of accessible links. The use of the partial structural information to reduce data amounts is also valuable discussion.

Because heterogeneous property is shared by many complex networks and there is a great need to ascertain network topology, this work aims to improve the compressed sensing based method by significantly reducing the data requirement for reconstructing heterogeneous networks. Specifically, two methods are proposed, the conflict-based method (CBM) and the element elimination method (EEM), where the former can remarkably reduce data amounts without relying on partial accessible links and the latter employs the partial structural information to reduce data amounts. The combination of the two methods can further reduce the required data compared to each the separate use of method. The two methods are validated using two representative evolutionary games occurring in scale-free networks, where it is assumed that individual information is measurable and an attempt is made to decode the network structure from measurable information. The results demonstrate that both methods and their incorporation indeed significantly reduce the data requirements compared to the original compressed sensing based method. Regarding the cost of accessing data, especially for biological systems, the efficient approaches presented here could have practical importance and potential applications in a wide range of fields.

## Methods

Two evolutionary games are employed that occur in complex networks to demonstrate how to implement the network reconstruction method. The evolutionary prisoner’s dilemma game (PDG) [28] has been a paradigm to understand cooperation among selfish individuals in nature and society [18, 20]. In the past decade, much interest has been given to the PDG on complex networks with focus on how network structure affects cooperation. Based on the PDG, many mechanisms have been proposed to facilitate cooperation, among which costly punishment [29, 30] as a representative altruistic behavior has been explored intensively. By incorporating the costly punishment mechanism, the PDG can be extended to a three-strategy game, called the cooperation-defection-punishment game (CDP).

In the networked PDG, each node is occupied by a player. At each round, a player can choose one of the two strategies (S): cooperation (C) or defection (D), which can be denoted as **S**(*C*) = (1, 0)^*T*^ and **S**(*D*) = (1, 0)^*T*^, respectively. For the CDP, there are three selectable strategies (S): cooperation (C), defection (D) and costly punishment (P), which can be denoted as **S**(*C*) = (1, 0, 0)^*T*^, **S**(*D*) = (0, 1, 0)^*T*^ and **S**(*P*) = (0, 0, 1)^*T*^, respectively. The profit of a player is determined by her/his strategy and the strategy of the co-player, according to a fixed payoff matrix. Without the loss of generality, two frequently used payoff matrices are used for the PDG [28] and CDP [29, 30], as follows:

$$P_{\text{PDG}}=\left(\begin{array}{cc} 1 & 0\\ 1.2 & 0 \end{array}\right)\;\;\;\;\text{and}\;\;\;\;P_{\text{CDP}}=\left(\begin{array}{ccc} 2 & -2 & -5\\ 4 & \;\;0 & -3\\ 2 & -2 & -5 \end{array}\right).$$ (1)

The profit gained by player *i* from playing with player *j* can be calculated by $S_{i}^{T}PS_{j}$. In each round, any player *i* plays the game (PDG or CDP) with their direct neighbors, and the total payoff G_*i*_ can be calculated by

$$G_{i}=\sum_{j\in \Gamma_{i}}S_{i}^{T}PS_{j},$$ (2)

where **S**_*i*_ and **S**_*j*_ are the strategies of player *i* and player *j*, respectively, Γ_*i*_ represents the set of neighbors for player *i*, and *P* = *P*_PDG_ if the players participate in PDG; otherwise, *P* = *P*_CDP_ if the players participate in CDP. After each round, the players update their strategies by learning from their neighbors. Specifically, the Fermi rule is used in the simulations, which can be described as follows: player *i* randomly selects one of her/his neighbors, e.g., *j*, and takes over *j*’s strategy with probability

$$W\;\left(S_{i}\leftarrow S_{j}\right)=\frac{1}{1+\exp[\left(G_{i}-G_{j}\right)/\kappa ]},$$ (3)

where *κ* represents the noise amplitude. In all simulations, based on existent investigations in the literature, *κ* = 0.1. During the evolution of PDG (or CDP), the time series of the strategies and he payoffs of all players are recorded.

In general, the problem of reconstructing complex networks can be converted into a sparse signal reconstruction problem, which can be addressed by using a compressed sensing approach. Specifically, compressed sensing aims at reconstructing the sparse vector **X** ∈ *R* ^*N*^ in the form **Y** = Φ ⋅ **X**, where **Y** ∈ *R* ^*M*^, and Φ is a *M* × *N* matrix. The sparse vector can be reconstructed by solving the following convex-optimization problem [11]:

$$\min∥X{∥}_{1}\text{subject}\;\text{to}\;Y=\Phi \cdot X,$$ (4)

where $∥X{∥}_{1}=\sum_{i=1}^{N}\left|X_{i}\right|$ is the *L*_1_ norm of vector **X** and matrix Φ satisfies the restricted isometry property [11–16]. One of the main advantages of compressed sensing is that the number of measurements is much less than the length of an unknown vector, that is, *M* ≪ *N*.

The relationship between the strategies and payoffs of each player is the key to reconstructing networks of the evolutionary games based on compressed sensing. The payoff of player of player *i* can be expressed as

$$G_{i}\left(t\right)=\sum_{j=1,j\neq i}^{N}a_{ij}F_{ij}\;\left(t\right),$$ (5)

where *a*_*ij*_ = 1 if player *i* and *j* are connected and *a*_*ij*_ = 0; otherwise, $F_{ij}\left(t\right)=S_{i}^{T}\left(t\right)\cdot P\cdot S_{j}\left(t\right)$ is the virtual payoff, which is exclusively determined by the strategies of *i* and *j*. If and only if *i* connects with *j*, the virtual payoff will become real payoff and gained by *i*. By measuring the strategies and payoffs of *M* accessible time instances *t*_1_, …, *t*_*M*_, Eq (5) can be expressed in the following matrix form:

$$Y_{i}=\Phi_{i}\cdot X_{i},$$ (6)

Where the virtual-payoff matrix Φ_*i*_, payoff vector **Y**_*i*_ and neighboring vector **X**_*i*_ can be written as

$$\Phi_{i}=\left[\begin{array}{cccc} F_{i1}\left(t_{1}\right) & F_{i2}\left(t_{1}\right) & \cdots & F_{iN}\left(t_{1}\right)\\ F_{i1}\left(t_{2}\right) & F_{i2}\left(t_{2}\right) & \cdots & F_{iN}\left(t_{2}\right)\\ \vdots & \vdots & \vdots & \vdots \\ F_{i1}\left(t_{M}\right) & F_{i2}\left(t_{M}\right) & \cdots & F_{iN}\left(t_{M}\right) \end{array}\right],$$ (7)

$$Y_{i}={\left[G_{i}\left(t_{1}\right),G_{i}\left(t_{2}\right),\cdots G_{i}\left(t_{M}\right)\right]}^{T},$$ (8)

$$X_{i}={\left[a_{i1},a_{i2},\cdots ,a_{iN}\right]}^{T}.$$ (9)

Because the virtual-payoff matrix Φ_*i*_ and the payoff vector **Y**_*i*_ can be immediately obtained from the time series of the strategies of all players and the payoffs of player *i*, the neighboring vector of player *i* can be uncovered based on compressed sensing. Because of the natural scarcity of the neighboring vector **X**_*i*_ in complex networks, only a small amount of data is sufficient to reconstruct **X**_*i*_, taking full advantage of the compressed sensing method in sparse signal reconstruction. Similarly, the neighboring vectors of all of the other players can be inferred, yielding the adjacency matrix *A* = [**X**_1_, **X**_2_, ⋯, **X**_*N*_] by assembling the neighboring vectors of all players. It is noteworthy that only one set of data is shared when reconstructing the neighboring vectors of different nodes, enabling the sparse data requirement.

However, the compressed sensing based method may not adequately function with respect to scale-free networks because of the existence of a small number of hubs that require much more data to reconstruct their neighbors due to their very high node degrees. Thus, the total data for fully reconstructing the entire network is determined by the hubs, and the hubs become the cask short board. This implies that it is likely to considerably reduce the data requirement for reconstructing the entire network if the relatively large amount of data for tackling a small number of hubs can be reduced. Therefore, the purpose is to propose a method to more efficiently reconstruct the neighboring vector of hubs to improve the compressed sensing based method for application on heterogeneous networks.

The improved method can be realized for undirected networks. The majority of nodes in scale-free networks are small-degree nodes, for which relatively small amounts of data are sufficient. By contrast, the data amount adequate for small-degree nodes is insufficient for hubs, accounting for the reconstruction errors of hubs. The key lies in how to identify the reconstruction errors and how to correct the errors. The tasks can be accomplished by exploiting the reconstruction conflict between hubs and their neighbors. Specifically, for an ingredient *a*_*ij*_ in the reconstructed vector **X**_*i*_ of *i* and *a*_*ji*_ in *j*, *a*_*ij*_ supposes to be equal to *a*_*ji*_ for accurate reconstructions. However, the situation may exist of *a*_*ij*_ ≠ *a*_*ji*_ because of inadequate data-induced reconstruction errors, especially between hubs and their neighbors. Thus, the frequency of encountering conflict allows hubs to be identified. After the inference of hubs, the reconstruction results of smaller-degree nodes can be used to replace those of hubs, which can effectively reduce the data amount for achieving accurate reconstruction. The conflict-based method (CBM) can be implemented by the following three steps:

1. Assign a threshold *λ*, if *a*_*ij*_ > *λ*; let *a*_*ij*_ = 1, deeming the prediction of link from *i* to *j* exists; otherwise, *a*_*ij*_ = 0.
2. If *a*_*ij*_ ≠ *a*_*ji*_, let *δ*_*ij*_ = 1; otherwise, *δ*_*ij*_ = 0. The conflict frequency of player *i* can be defined as $C_{i}=\sum_{j=1,j\neq i}^{N}\delta_{ij}$.
3. For player *i* and player *j*, if *C*_*i*_ > *C*_*j*_, replace *a*_*ij*_ with *a*_*ji*_; otherwise, the value of *a*_*ij*_ is unchanged.

**Fig 1:**
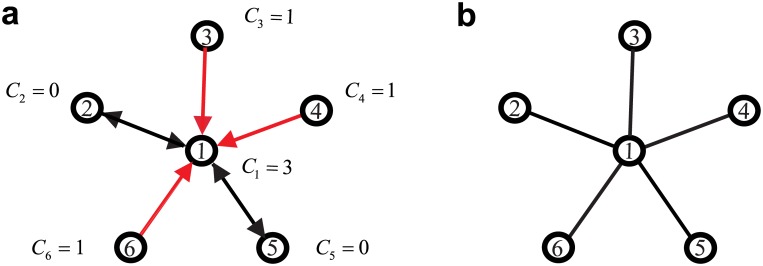
Schematic illustration of the location of the hubs from the conflicts. A star network is used as an example. The degrees of the hub node 1 and other nodes are 5 and 1, respectively. **a**, The schematic illustration is a possible compressed sensing result. The two-way arrows show that no conflict exists, and the one-way arrows indicate that conflict occurred. The conflict frequency is 3 for the hub and 0 or 1 for the other nodes. The conflict frequency of the hub is large than the other nodes. Thus, whether there are links between the hub and the others is determined by the other nodes rather than the hub. **b**, The reconstructed sample graph after implementing the conflict-based method (CBM).

Fig 1 provides an intuitive example of a counting conflict for each node and demonstrates why hubs frequently accompany more conflicts.

If a fraction of links in a network is accessible in advance, the partial structural information may be used to reduce the data amount for precise reconstruction. Specifically, this technique is called the Element Elimination Method (EEM) and is described as follows. Assume that a known fraction of the connections of node *i* exists, denoted by *Π*_*i*_, Eq 5 can then be rewritten as

$$G_{i}\left(t\right)=\sum_{j\in \Pi_{i}}a_{ij}F_{ij}\;\left(t\right)+\sum_{j\notin \Pi_{i}}a_{ij}F_{ij}\;\left(t\right),$$ (10)

where the first term in the right side are the payoffs of *i* gained from playing with already known neighbors *Π*_*i*_ and the second term is the virtual payoff from playing with unknown neighbors. After subtracting ∑_*j* ∈ *Π*_*i*__ *a*_*ij*_ *F*_*ij*_(*t*) on the both sides of Eq 5, the unknown connections of *i* can be reconstructed by optimizing the solution of the following equation using the compressed sensing approach:

$$G_{i}\left(t\right)-\sum_{j\in \Pi_{i}}a_{ij}F_{ij}\;\left(t\right)=\sum_{j\notin \Pi_{i}}a_{ij}F_{ij}\;\left(t\right).$$ (11)

Consider a simple example with only one accessible link between player *i* and *x*. The payoff and adjacency matrices can be written as

$$Y_{i}^{\prime }={\left[G_{i}\left(t_{1}\right)-a_{ix}F_{ix}\left(t_{1}\right),G_{i}\left(t_{2}\right)-a_{ix}F_{ix}\left(t_{2}\right),\cdots G_{i}\left(t_{M}\right)-a_{ix}F_{ix}\left(t_{M}\right)\right]}^{T},$$ (12)

$$X_{i}^{\prime }={\left[a_{i1},a_{i2},\cdots ,a_{i,x-1},\;0\;,a_{i,x+1},\cdots ,a_{iN}\right]}^{T}.$$ (13)

After this operation, the unknown connections of *i* can be reconstructed by optimizing the solution of the following equation using the compressed sensing approach:

$$Y_{i}^{\prime }=\Phi_{i}\cdot X_{i}^{\prime },$$ (14)

Note that vector $X_{i}^{\prime }$ is sparser than the original vector **X**_*i*_ without using EEM, accounting for the requirement of less amounts of data based on compressed sensing.

Moreover, it is expected that the incorporation of CBM and EEM can offer a better reconstruction compared to using each method separately.

## Results

The PDG and CDP are simulated on two types of scale-free networks, Barabási-Albert networks (BA) and a static model (SM), whose degree distribution follows the power law *P*_*D*_(*k*)∼*k* ^−*γ*^. For the BA networks, the power index is *γ* = 3. In the SM network, the exponent of the power-law degree distribution is adjustable; without the loss of generality, it is set to 2.25.

**Fig 2:**
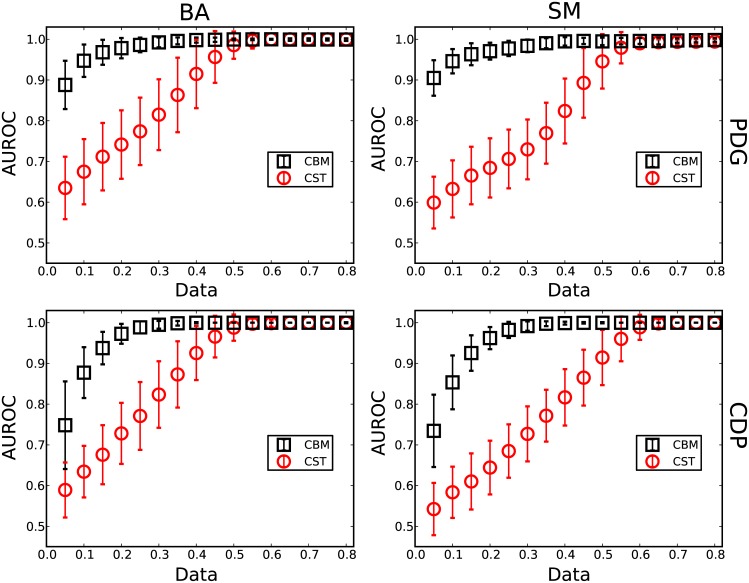
AUROC of reconstructing heterogeneous networks. The values of AUROC of reconstructing the maximum degree node of two types of scale-free networks, Barabási-Albert networks (BA) and the static model (SM), based on the time series obtained from two evolutionary games, the basic prisoner’s dilemma game (PDG) and the cooperation-defection-punishment game (CDP). The red squares denote the results only with compressed sensing theory (CST). The blue circles denote the results with the conflict-based method (CBM) based on compressed sensing. The network size *N* is 100, and the average degree 〈*k*〉 is 4. Each data point is obtained by averaging over 100 independent realizations. The error bars denote the standard deviations.

**Fig 3:**
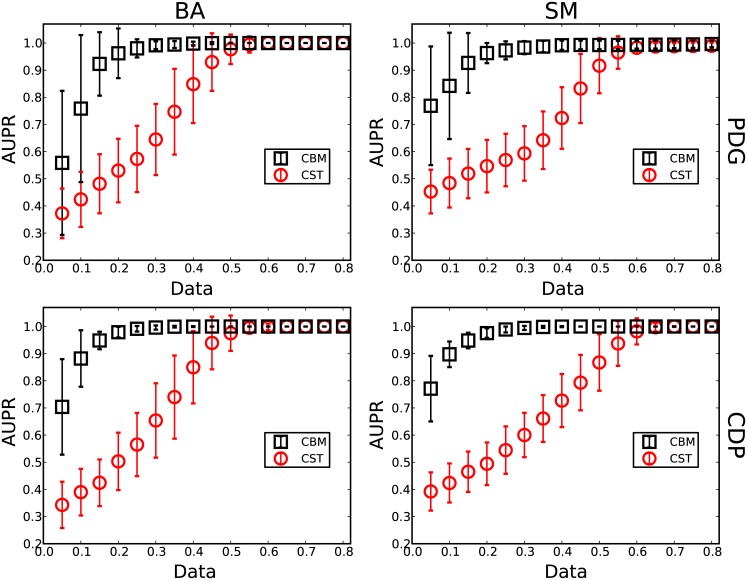
AUPR of reconstructing heterogeneous networks. The values of AUPR of reconstructing the maximum degree node of two types of scale-free networks, BA and SM, based on the time series obtained from two evolutionary games, PDG and CDP. The red squares denote the results with CST. The blue circles denote the results with CBM based on compressed sensing. The other parameters are identical to Fig 2.

For the two evolutionary games, the strategies and payoffs of players are recorded in each round to apply the method to reconstruct networks with different amounts of data (Data ≡ *M*/*N*, where *M* is the number of accessible time instances in the time series). Two standard indices are applied, the area under the receiver operating characteristic curve (AUROC) and the area under the precision-recall curve (AUPR) to qualify the reconstruction performance of this method. In particular, before calculating the AUROC and the AUPR at different thresholds, the elements are adjusted in the predicted adjacency matrix using the conflict-based method. Because the amount of data needed to fully reconstruct the heterogeneous networks are mainly determined by hubs, the method is validated based on the performance of reconstructing the neighbors of the maximum degree node. Figs 2 and 3 show the results of reconstructing the BA and SM networks for the two types of evolutionary games. It is clear that the conflict-based method (CBM) can greatly increase the reconstruction accuracy and decrease the amount of data. For example, as shown in Fig 2, the amount of data needed for an accurate reconstruction of the BA network for PDG is approximately 60% without CBM. In contrast, after applying the CBM, the amount of data decreases to roughly 30% and 40%, achieving a 50% decrease in the data requirement. For the SM networks, the amount of data decreases to approximately 40% for an accurate reconstruction with CBM. Even with a small amount of data, e.g., Data = 0.1, the reconstruction accuracy is still significantly improved. These results demonstrate that heterogeneous networks can be efficiently inferred from limited time series using this method.

**Fig 4:**
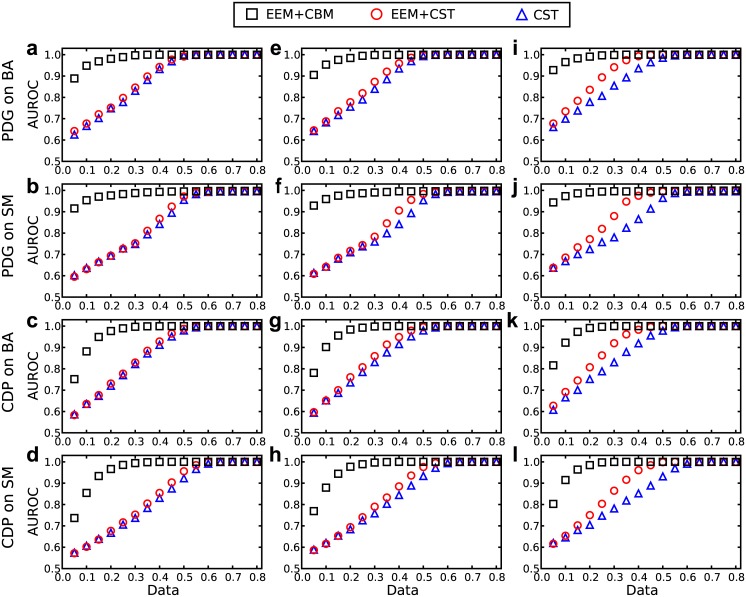
AUROC of reconstructing heterogeneous networks with a part of accessible connections. The values of AUROC of reconstructing the maximum degree node of two types of scale-free networks, BA and SM, based on the time series obtained from two evolutionary games, PDG and CDP, with 10%(**a-d**), 20%(**e-h**) and 40%(**i-l**) of the accessible connections. The red circles denote the results of removing those accessible connections before reconstruction with the CBM based on compressed sensing. The black hexagons denote the results of removing those accessible connections before reconstruction just by compressed sensing. The green diamonds denote the results of reconstructing networks only by compressed sensing without removing those accessible connections. The other parameters are identical to Fig 2.

**Fig 5:**
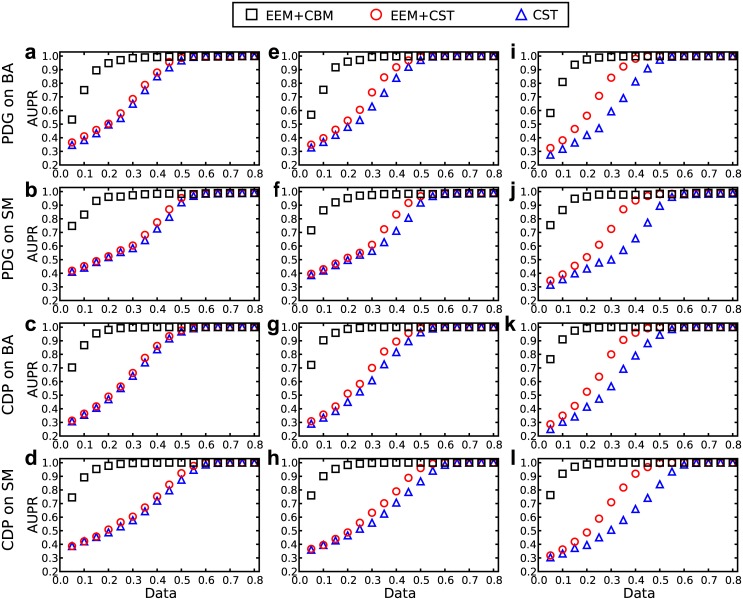
AUPR of reconstructing heterogeneous networks with a part of accessible connections. The values of AUPR of reconstructing the maximum degree node of two types of scale-free networks, BA and SM, based on the time series obtained from two evolutionary games, PDG and CDP, with 10%(**a-d**), 20%(**e-h**) and 40%(**i-l**) of the accessible connections. The other parameters are identical to Fig 4.

To validate the element elimination method (EEM), it is assumed that a fraction of connections are accessible in advance. Figs 4 and 5 show the results of reconstructions with respect to 10%, 20% and 40% of the available connections in the networks. When the proportion of accessible connections is small, e.g., 10%, there is no significant improvement if EEM is used when compared with compressed sensing. When the proportion of accessible connections is large, e.g., 40%, the time series needed to reconstruct networks decreases by approximately 10% if using EEM. Considering the accessible information about connections helps to reconstruct the complex networks, and the combination of CBM and EEM is quite effective in increasing accuracy and decreasing data requirements.

## Discussion

In many complex systems, the amount of available data may be sporadic and limited with respect to network size, raising the need to develop efficient approaches to reconstructing complex networks with low data requirements. Despite recent advances in network reconstruction based on compressed sensing, larger amounts of data are required for reconstructing heterogeneous networks than for homogeneous networks. However, full advantage is not taken of compressed sensing in sparse signal reconstruction. Two methods are proposed, the conflict-based method and the element elimination method, to greatly improve the efficiency of compressed sensing based methods in reconstructing heterogeneous networks. The two methods have been validated by taking two types of scale-free networks as examples, demonstrating that the amount of data required for achieving accurate reconstruction is indeed greatly reduced compared to the original compressed sensing-based method. The incorporation of the two methods offers better reconstruction performance than using each method separately. The prevalence of heterogeneous networks in nature and society allows these approaches to have potential applications in a wide range of fields. Note that although CBM and EEM are not limited to reconstructing heterogeneous, the performance of using CBM and EEM for reconstructing homogeneous networks is not as good as that of heterogeneous networks, because of the lack of cask short board (high degree nodes). Thus our methods in principle are applicable to heterogeneous networks with hubs. Meanwhile, this work raises some open questions, the answer to which can further deepen the understanding of the network reconstruction problem. First, although the efficiency and accuracy of reconstructing network topology has been remarkably improved, it is still challenging to determine how link weights can be more exactly inferred. Second, it is hard to figure out how the two methods can be extended to directed networks, especially CBM, because conflict can not be defined for directed networks. Third, it is a fundamental problem to determine the application of this method beyond social interaction networks, such as in gene regulation networks, protein-protein interaction networks and brain networks. Nevertheless, this work opens new methods to reconstruct heterogeneous networks in a more efficient manner, and it is expected to stimulate further efforts to pursue better approaches to address the inverse problem with a broader application scope.
